# Decoding Overlapping Lower-Limb Afferent Pathways from Human Epidural Spinal Recordings

**DOI:** 10.21203/rs.3.rs-10348238/v1

**Published:** 2026-07-22

**Authors:** Alexander G. Steele, Milton O. Candela, Gracie J. Hufft, Amanda Howes-Keith, Catherine Martin, Jeonghoon Oh, Amir H. Faraji, Dimitry G. Sayenko

**Affiliations:** 1 Center for Neuroregeneration, Houston Methodist Research Institute, Houston, Texas, United States of America; 2 Department of Neurosurgery, Houston Methodist, Houston, Texas, United States of America

## Abstract

Restoration of dynamic motor function following neurological injury increasingly relies on adaptive neuroprostheses, which require real-time sensory feedback to continuously adjust to a user’s physical state. However, it remains unknown whether distinct afferent activity can even be decoded from highly overlapping, volume-conducted epidural fields. This challenge is particularly pronounced in the lumbosacral enlargement, where common fibular (CFN) and tibial nerve (TN) afferents converge extensively, producing highly similar cord dorsum potential (CDP) topographies. Here, we demonstrate for the first time in humans that clinical-grade lumbosacral epidural paddle arrays capture sufficient fine-scale spatiotemporal structure to decode these overlapping inputs. Using a 32-contact array and peripheral nerve stimulation, we constructed a 42-dimensional feature space capturing distributed amplitudes, field geometry, and waveform morphology. A support vector machine decoded four distinct afferent classes (left and right CFN and TN) with a median accuracy of 90.9% ± 0.3%. Shapley Additive Explanations revealed decoding was driven by contact-level voltage patterns and temporal waveform complexity, while the geometric features contributed minimally. These afferent-specific signatures persisted even at sub-motor threshold stimulation intensities. By utilizing standard clinical arrays, this approach provides a pathway toward rapid deployment of interpretable closed-loop neuromodulation, avoiding the surgical risks of penetrating or peripheral interfaces.

## Introduction

**Figure 1 –:**
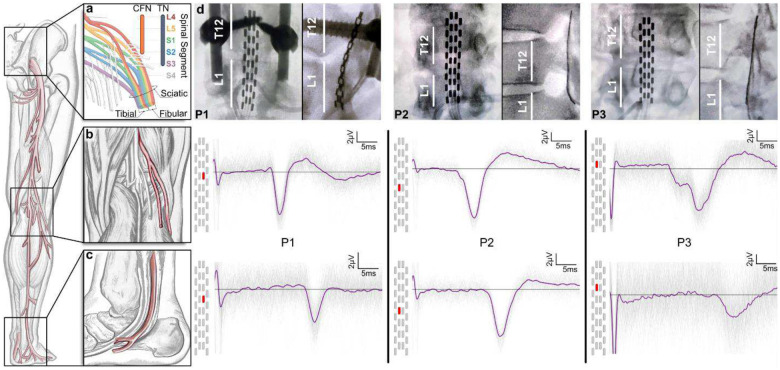
Experimental paradigm and epidural interface. **a.** The sciatic nerve is composed of projections from multiple lumbosacral spinal segments **b.** ultimately bifurcating into the common fibular and tibial nerves while maintaining extensive segmental overlap. Transcutaneous electrical stimulation was applied to the **b**. common fibular (CFN) and **c.** tibial (TN) nerves to elicit ascending sensory volleys. **d.** Anteroposterior (left) and lateral (right) fluoroscopy confirms the surgical placement of a 32-contact epidural paddle array spanning the T12 to L1 vertebral levels across three participants (P1–P3) with complete spinal cord injury. The lateral views verify the dorsal positioning of the array within the epidural space. Solid white lines denote the relevant vertebral body margins. Below the fluoroscopic images, representative single-trial (gray) and trial-averaged (purple) epidural cord dorsum potentials (CDPs) elicited from supra-motor threshold (Supra-MT) stimulation of the right CFN (top) and right TN (bottom) demonstrate robust, stimulus-locked responses across participants (0–40ms post-stimulus).

Epidural spinal stimulation (ESS) has emerged as a powerful neuromodulatory and neuroprosthetic therapy, yet its current clinical application remains largely open-loop^1–4^. A critical next step for the field is the development of bidirectional interfaces capable of sensing spinal cord activity and adapting stimulation in real time^5–8^. To achieve this, understanding the state of the human spinal cord’s sensory circuitry and the spinal targets of incoming afferent signals is essential. In the lumbosacral enlargement, common fibular (CFN) and tibial (TN) nerve afferents converge extensively, sharing overlapping dorsal root entry zones and segmental projections (Fig. 1a)^9–11^. This convergence produces highly similar cord dorsum potential (CDP) topographies when recorded epidurally, limiting the ability to infer the source of distinct peripheral inputs from epidural recordings^12–15^. Disentangling these overlapping representations remains a central bottleneck for bidirectional neuroprosthetic systems that depend on reliable sensory state estimation^16–18^.

A range of neural interface strategies have been proposed to access afferent information. Penetrating approaches, including intraneural and intraspinal electrodes, can achieve high spatial selectivity but are constrained by long-term stability challenges and sensitivity to micromotion induced by physiological spinal movement^19,20^. Peripheral nerve interfaces, such as cuff electrodes, provide a comparatively less invasive alternative but require additional surgical exposure of individual nerves, introducing increased hardware complexity, additional points of failure, and surgical burden to the individual^21–23^.

In contrast, epidural spinal cord arrays are already in widespread clinical use for neuromodulation, offering a stable and minimally invasive interface to spinal pathways without penetrating neural tissue^24–26^. However, it remains an open question whether these highly overlapping, volume-conducted epidural fields contain sufficient fine-scale spatiotemporal information to resolve individual afferent sources.

Here, we test whether clinical-grade high-density epidural paddle array recordings contain sufficient structured information to decode overlapping lower-limb afferent inputs. Operating on a 42-dimensional spatiotemporal feature space derived from a 32-contact array, we evaluate a four-class nonlinear support vector machine against a linear baseline (Linear Discriminant Analysis) to classify CFN and TN inputs bilaterally. Furthermore, to ensure interpretability, we apply SHapley Additive Explanations (SHAP) to attribute model decisions directly to specific spatial and temporal features^27,28^. By providing the first-in-human demonstration of reliable afferent decoding from epidural signals, this work establishes a critical computational framework. By utilizing the anatomical coverage of standard clinical arrays, this approach provides a potential pathway toward clinically deployable, source-aware bidirectional spinal interfaces.

## Results

The primary objective of this study was to determine whether CDPs evoked by spatially overlapping peripheral afferents could be reliably distinguished using epidural recordings. Across participants, a cubic kernel SVM operating on a 42-dimensional spatiotemporal feature space decoded overlapping lower-limb afferent inputs from epidural CDPs with high cross-validated accuracy (90.9% ± 0.3%).

### Participant information

All experimental procedures were approved by the Houston Methodist Research Institute Institutional Review Board (IRB: PRO00037483) under an Investigational Device Exemption (IDE: G2303171). The study is registered at ClinicalTrials.gov (NCT06213012). Two female and one male participant were enrolled in the study: Participant 1 (P1, 34 years old), Participant 2 (P2, 37 years old), and Participant 3 (P3, 36 years old), all with chronic, traumatic, motor and sensory complete thoracic spinal cord injury (SCI) at levels T8, T7, and T12, respectively. Injuries were sustained approximately 7 (P1), 21 (P2), and 2 (P3) years prior to enrollment and were classified as American Spinal Injury Association Impairment Scale (AIS) A according to the International Standards for Neurological Classification of Spinal Cord Injury (ISNCSCI). Participants provided written informed consent and were compensated for time and travel.

### Surgical procedures and epidural implantation

The lumbosacral spinal cord receives extensive, overlapping sensory projections from the sciatic nerve (Fig. 1a) and its primary distal branches, the common fibular (Fig. 1b) and tibial (Fig. 1c) nerves. To capture epidural recordings over this convergence zone, we recruited participants who were independently undergoing surgical implantation of a standard clinical-grade, 32-contact epidural paddle array (CoverEdge X 32; size: 67mm × 10mm, Boston Scientific, USA) for spinal neuromodulation therapy. Access to the epidural space was achieved via a laminectomy at the L2 vertebral level. The paddle array was then advanced rostrally and positioned over the dorsal aspect of the lumbosacral enlargement. Final electrode placement was confirmed via a combination of intraoperative fluoroscopy and electrophysiology, with the array consistently spanning the T12 and L1 vertebral levels across participants (Fig. 1d). To facilitate high-resolution electrophysiological data acquisition during the testing phase, the electrode leads were tunneled subcutaneously and temporarily externalized, allowing direct connection to the external recording amplifier.

### Experimental protocol

Participants were positioned supine and fully supported. Peripheral nerve evoked responses were elicited using transcutaneous electrical stimulation (2.3 cm surface electrodes; PALS, Axelgaard Manufacturing Co., Fallbrook, CA) in a cathode-proximal/anode-distal configuration^29–31^. The CFN was stimulated inferior to the fibular head (Fig. 1b), and the TN was stimulated posterior to the medial malleolus (Fig. 1c)^10,11^. Stimulation sites were determined by using single-pulse mapping to identify locations eliciting consistent motor responses at the lowest achievable threshold.

For each nerve (left/right CFN and TN), 200 monophasic pulses (200 μs, 2.1 Hz) were delivered using an isolated constant-current stimulator (DS8R, Digitimer, UK). Conditions were randomized and separated by ≥5 min rest. Stimulation was delivered at sub-motor threshold (Sub-MT; 0.8×MT), motor threshold (MT; 1.0×MT), and supra-threshold (Supra-MT; 1.2×MT). Epidural CDPs were recorded from the implanted paddle array using custom-built hardware and a 32-channel actiCHamp amplifier sampling at 25 kHz (actiCHamp Plus, Brain Products, NC; CMRR >100 dB; gain 1000; resolution 0.04883 μV/bit).

### Spatial co-registration and anatomical normalization

To ensure anatomical consistency across subjects and correct for fluoroscopic projection distortion, paddle geometry was reconstructed using a control-point-based spatial transformation.

**Figure 2 –:**
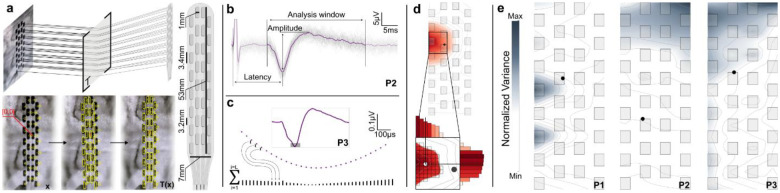
Spatiotemporal feature extraction and anatomical normalization. **a.** To standardize spatial comparisons across participants and correct for fluoroscopic projection distortion, empirical contact coordinates (left) were mapped to an idealized paddle template (right) using a nonlinear spatial transformation matrix (T), anchored to the T12 inferior endplate. **b.** Epidural cord dorsum potentials (CDPs) were analyzed within a 10–40ms post-stimulus window to extract primary features, including peak-to-peak amplitude and latency to the peak response while eliminating the low-latency stimulation artifact. **c.** Waveform complexity was quantified by calculating the line length (the sum of absolute differences between consecutive voltage measurements) across the analysis window. **d.** This normalized coordinate system enabled the extraction of geometric features from the interpolated voltage fields, including the spatial center of mass (white marker) and the activation area (area exceeding 90% of global maximum). **e.** Representative spatial heatmaps of the normalized signal variance across the paddle array, calculated using a 40–50ms post-stimulus window across all conditions highlights the underlying noise distribution of the data.

Idealized contact coordinates were defined from known paddle geometry (1.0 mm × 3.2 mm contacts, 3.2 mm rostro-caudal spacing, 1 mm mediolateral spacing). Empirical contact positions were extracted from fluoroscopic images (Fig. 2a) and mapped to this template using a nonlinear spatial transformation

(1) $$\left[X_{c},Y_{c}\right]=T\left({x^{\prime }}_{c},{y^{\prime }}_{c}\right)$$

where for each contact *c*, (*x’*_*c*_*, y’*_*c*_) represent the initial image-space coordinates, [*X*_*c*_*, Y*_*c*_] are the anatomically normalized coordinates in millimeters, and *T* defines a nonlinear transformation mapping this relationship. The resulting coordinate system was anchored ([0, 0]) to the anatomical midline of the T12 inferior endplate, standardizing spatial comparisons across participants while preserving true epidural geometry.

### Feature extraction

Each trial was represented by a 42-dimensional feature vector capturing spatial amplitude, geometric structure, and temporal morphology of CDPs within a physiologically relevant 10–40ms post-stimulus window.

Amplitude features consisted of peak-to-peak voltage values at each of the 32 epidural contacts (Fig. 2b), preserving the spatial footprint of the CDPs across the paddle array. To maintain continuity following local reference subtraction, missing values were reconstructed using natural neighbor interpolation^32,33^. Temporal and morphological features captured dynamic response properties, including latency (Fig. 2b) to peak voltage from the contact that elicited the largest peak-to-peak response over the array and waveform complexity quantified using line length (Fig. 2c; Supplementary Table 1).

Geometric features were derived from the interpolated CDP field over the paddle array constructed in the anatomically normalized coordinate space (Fig. 2d). These included peak voltage and its spatial location (x, y), hotspot area defined as the region exceeding 90% of the maximum voltage (V_max_), center of mass (x_CoM_, y_CoM_), and spatial dispersion, which was quantified as the amplitude-weighted standard deviation of spatial activation within the hotspot. Finally, to quantify the underlying noise over the array and identify any common bias, the normalized signal variance across the paddle array was calculated using a 40–50ms post-stimulus window across all conditions (Fig. 2e).

### Anatomical convergence and spatial overlap of epidural evoked potentials

**Figure 3 -:**
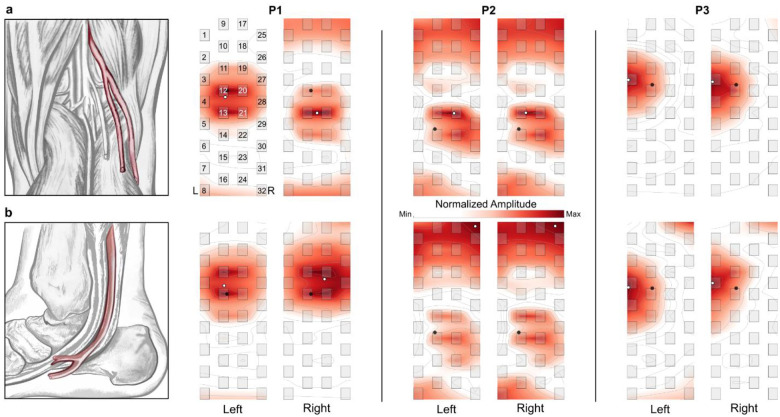
Spatial topography of overlapping epidural evoked potentials. Interpolated spatial voltage maps (peak-to-peak amplitude averaged over the included repetitions) across the 32-contact epidural array during supra-motor threshold stimulation of the **a.** common fibular and **b.** tibial nerves for all three participants during stimulation of the left (left maps) or right nerve (right maps). To facilitate subsequent contact-level analysis and establish anatomical orientation, the array’s contact numbering scheme (1–32) and lateral boundaries (L: Left, R: Right) are superimposed on the top-left array (P1). Despite originating from distinct peripheral nerves, the epidural spatial footprints exhibit extensive anatomical convergence and volume-conducted overlap, particularly in P1 and P3. The anatomical location of the T12 inferior endplate is denoted by the black marker, and the spatial center of mass for each response is denoted by the white marker and was calculated using the area of activation that exceeded 90% of the maximum response over the array.

To evaluate the macroscopic representation of distinct lower-limb afferents, we first mapped the peak-to-peak voltage amplitudes of CDPs evoked by supra-MT stimulation (Fig. 3) of the common fibular (Fig. 3a) and tibial (Fig. 3b) nerves across the 32-contact epidural array. Visual inspection of the interpolated spatial topographies revealed extensive volume-conducted overlap and profound anatomical convergence. Within each individual participant, the epidural spatial footprints for both nerve targets exhibited highly overlapping, near-identical topographies, with particularly severe spatial blending observed in participants P1 and P3. This high degree of intra-subject spatial convergence demonstrates that gross topological features and single-metric visual inspection are insufficient to reliably separate these distinct afferent pathways.

### Decoding of overlapping afferent inputs from epidural fields

A total of 6,000 single-trial CDP recordings were acquired across all stimulation intensities (2,000 trials per intensity condition). Following artifact rejection (10.2% overall exclusion), the dataset yielded 1,818 retained trials at the supra-motor threshold (Supra-MT; 9.1% rejection rate), 1,800 retained trials at the motor threshold (MT; 10.0% rejection rate), and 1,771 retained trials at the sub-MT (11.5% rejection rate). A series of Chi-square goodness-of-fit tests confirmed no evidence of differential exclusion by class within any intensity level, ensuring highly balanced datasets for all classification models (Supra-MT: *χ*^2^(3) = 0.152, *p* = 0.985; MT: *χ*^2^(3) = 0.164, *p* = 0.983; Sub-MT: *χ*^2^(3) = 1.375, *p* = 0.711).

**Figure 4 –:**
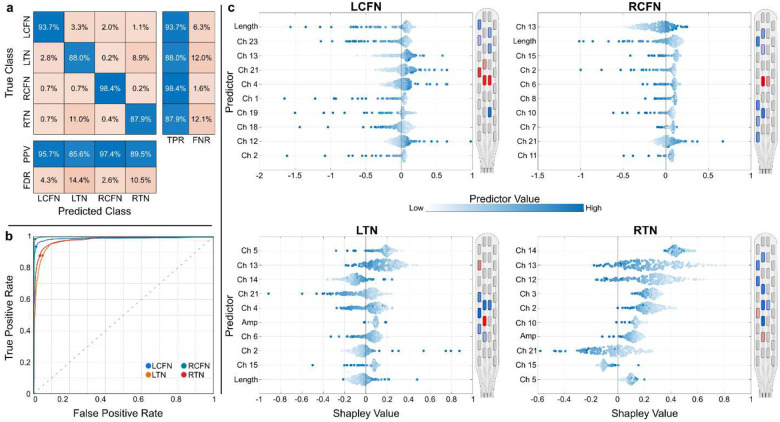
Nonlinear decoding and feature attribution of overlapping afferent sources. A SVM utilizing a 42-dimensional spatiotemporal feature space successfully discriminated highly convergent afferent inputs. **a**. Cross-validated, row-normalized confusion matrix for supra-motor threshold (Supra-MT) stimulation demonstrating 92.0% overall classification accuracy. Margins display the corresponding positive predictive values (PPV) and false discovery rates (FDR) by column, alongside true positive (TPR) and false negative rates (FNR) by row. To account for partition variance and ensure robustness, repeated cross-validation (100 iterations) was performed, establishing a true median accuracy of 90.9% ± 0.3% across the dataset. **b.** Receiver operating characteristic (ROC) curves confirming high class separability across all four neural targets (AUC: 0.975–0.998). **c.** SHAP (SHapley Additive Explanations) summary plots identifying the primary drivers of model classification, where individual points represent single trials. Adjacent to each plot, a corresponding schematic of the 32-contact array maps the anatomical distribution and directionality of the top predictive channels. Contacts are color-coded by their contribution to a positive classification: red indicates that high feature values drive the prediction, while blue indicates that low feature values drive the prediction. Color saturation denotes feature prominence, with darker shades corresponding to higher predictive weight. Overall, contact-level peak-to-peak amplitudes (e.g., Ch 13, Ch 14) and temporal waveform complexity (Line Length) carried the highest predictive weight, demonstrating that afferent identity is encoded in distributed, fine-scale spatiotemporal gradients rather than derived geometric features.

Using the pooled dataset, the SVM successfully decoded overlapping lower-limb afferent inputs from supra-MT evoked CDPs. To ensure the robustness of this performance against partition variance, models were evaluated using 100 iterations of 10-fold cross-validation. Using the full 42-dimensional spatiotemporal feature space, the model achieved a median cross-validated accuracy of 90.9% ± 0.3%. A representative model, demonstrating 92.0% overall classification accuracy, is depicted in Fig. 4a. Despite the extensive anatomical convergence of these projections within the lumbosacral enlargement, CFN and TN inputs from both sides of the body were reliably separated. Class-specific performance in this representative model remained robust, yielding true positive rates of 93.7% and 98.4% for the left and right CFN, respectively, alongside 88.0% and 87.9% for the left and right TN (Fig. 4a). Furthermore, ROC analysis confirmed strong, nonlinear separability across all four neural targets, yielding AUC values ranging from 0.975 to 0.998 (Fig. 4b).

### Feature contributions

SHAP analysis revealed that decoding was primarily driven by the amplitudes of highly specific epidural contacts and temporal waveform complexity, rather than the geometric features of the overall field structure (Fig. 4c). Instead of relying on broad spatial gradients (e.g., a medial-lateral divide), the model consistently relied on the same subset of specific physical contacts, acting as spatial “anchors” for the decision boundary, but evaluated them in a class-dependent manner. For instance, contact 13 (Ch 13) emerged as a top-three predictor across all four nerve targets, yet its discriminative direction inverted depending on the class: high amplitude at Ch 13 strongly drove CFN classification, whereas low amplitude was highly predictive of TN. Temporal waveform complexity (Line Length) provided a critical secondary axis of class separation, though its utility was asymmetrical across targets. Low signal complexity consistently drove the classification of CFN, whereas high complexity strongly predicted LTN classification exclusively. Notably, Line Length dropped out of the top predictors entirely for RTN. This indicates that while the model utilizes spatiotemporal divergence for most targets, it relies largely on fine-scale spatial voltage patterns (specifically Ch 14 and Ch 13) to isolate RTN responses.

### Decoding robustness and intensity-dependent state-space expansion

**Figure 5 -:**
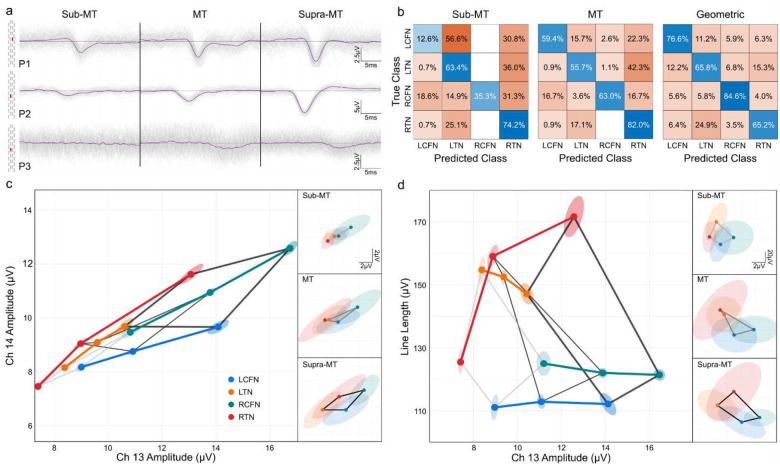
Decoding robustness and changes in spatiotemporal representations. Representative right tibial nerve CDPs **a.** evoked at sub-motor threshold (Sub-MT), motor threshold (MT), and supra-motor threshold (Supra-MT), shown during the analysis window (10–40ms), demonstrating a progressive attenuation of signal amplitude with decreasing stimulation intensity, a trend consistently observed across participants. Despite this degradation, the fixed Supra-MT trained SVM maintained classification accuracies of **b.** 46.0% at Sub-MT (left) and 65.0% at MT (middle), operating substantially above chance (25%) and indicating the persistence of afferent-specific structure. When the model was restricted exclusively to geometric spatial features (e.g., center of mass, area), the Supra-MT accuracy (right) collapsed to 73.0%, highlighting the model’s reliance on fine-scale voltage gradients. To further quantify feature changes, phase space representations of the **c.** spatial (Ch 13 vs. Ch 14) and **d.** spatiotemporal (Ch 13 vs. Line Length) divergence were plotted. Solid colored lines track the trajectory of each class manifold as stimulation intensity increases from Sub-MT (bottom-left points) to Supra-MT (top-right points). Grey and black lines represent the state-space boundaries separating the four classes at a given intensity. Colored ellipses in the main plots mark the standard error of the mean (SEM). For clarity, individual intensity manifolds are isolated in the subplots (right), where colored ellipses denote the wider one standard deviation probability density, illustrating how overlapping sub-threshold measurements structurally diverge into highly separable, source-specific representations at higher intensities.

To evaluate model robustness against signal degradation, we tested the supra-MT-trained SVM on CDPs evoked at lower stimulation intensities. As demonstrated by representative right tibial nerve CDPs (Fig. 5a), signal amplitude progressively attenuated at Sub-MT and MT when compared to Supra-MT. Despite this degradation, when the Supra-MT decision boundaries were applied to the lower-intensity pooled datasets, the model maintained classification accuracies of 46.0% and 65.0%, respectively (Fig. 5b). While degraded, these accuracies remain substantially above chance (25%), indicating that afferent-specific spatiotemporal structure persists even at stimulation intensities below motor threshold (i.e., Sub-MT).

To determine if this performance drop was due to an inherent loss of information or merely a shift in state space, we subsequently trained and evaluated independent models exclusively within each intensity tier (Supplementary Figure 1). Through repeated CV (100 iterations), models trained on the lower-intensity data successfully recovered classification performance, achieving median accuracies of 64.5% ± 0.6% (Sub-MT) and 81.6% ± 0.4% (MT). This demonstrates that afferent identity remains robustly encoded at sub-threshold levels, but the spatial topographies shift such that decision boundaries do not linearly generalize across intensities.

Conversely, to evaluate the model’s reliance on feature space resolution, the Supra-MT model was artificially restricted to rely exclusively on the derived geometric features (e.g., center of mass, total activation area). This restriction caused the overall median classification accuracy to collapse from 90.9% ± 0.3% (Fig. 4a) to 73.2% ± 0.6% (Fig. 5b, right). To evaluate this performance degradation, a paired permutation test (10,000 iterations) was applied to the out-of-fold trial predictions. This confirmed that the uncompressed spatiotemporal feature space provided significantly superior classification over the geometric features alone (P < 0.0001).

To visualize the mechanical basis of this feature reliance and intensity-dependent shifting, we mapped the physiological state space across stimulation intensities. Plotting spatial divergence (Fig. 5c) and spatiotemporal divergence (Fig. 5d) revealed that sub-threshold representations exist as a highly overlapping, indistinguishable core. As stimulation intensity increases, these overlapping pools do not simply scale in magnitude; they structurally diverge along distinct, nonlinear trajectories into highly separable, source-specific topological domains.

Finally, to establish a baseline for spatial separability and evaluate the necessity of nonlinear model architecture, we trained an LDA model on the full Supra-MT feature space (Supplementary Figure 2). While the linear baseline achieved a robust median classification accuracy of 80.2% ± 0.3%, it could not fully resolve the highly overlapping afferent representations compared to the cubic kernel SVM (90.9% ± 0.3%). This performance gap confirms that these volume-conducted epidural fields produce complex, nonlinearly separable boundaries.

## Discussion

To our knowledge, we provide the first-in-human demonstration that clinically deployed epidural arrays contain sufficient fine-scale spatiotemporal structure to decode highly convergent afferent pathways. Although epidural signals are routinely interpreted as aggregate evoked responses, our results demonstrate that CDPs preserve a recoverable, afferent-specific signature. Operating within a 42-dimensional spatiotemporal feature space, our SVM achieved a median cross-validated accuracy of 90.9% ± 0.3%, correctly resolving four distinct afferent classes (left and right CFN and TN) despite their extensive anatomical overlap within the lumbosacral enlargement. SHAP feature attribution and paired permutation testing confirmed that this performance arises from fine-scale, contact-level voltage patterns and temporal waveform complexity rather than gross amplitude differences. Critically, when the model was artificially restricted to conventional macroscopic representations (such as aggregate amplitude or centroid measures), classification accuracy collapsed significantly. This reveals that low-dimensional geometric features may compress and discard essential source information, whereas preserving uncompressed spatial gradients is needed for more accurate afferent discrimination. Furthermore, we found that these afferent-specific signatures persist at lower stimulation intensities; rather than an inherent loss of information, sub-threshold representations undergo nonlinear, intensity-dependent shifts in state space that remain decodable.

CDPs are traditionally evaluated using single-channel peak-to-peak amplitudes or latency along the rostrocaudal axis^1,34–36^. While geometric features, such as spatial center of mass, are routinely applied to decipher overlapping cortical or muscle fields, our data indicate that relying on these derived features is insufficient to separate highly convergent spinal afferents^37–40^. When we artificially restricted our model to rely exclusively on the derived geometric features, median classification performance degraded significantly to 73.2% ± 0.6% (P < 0.0001, paired permutation test). This stands in stark contrast to the 90.9% ± 0.3% median accuracy achieved when the uncompressed contact-level voltage structure was retained. Our SHAP analysis mechanistically explains this performance gap: decoding is driven primarily by fine-scale spatial voltage patterns across discrete epidural contacts rather than their global spatial summaries. By identifying that specific contacts, particularly those near the midline of the epidural array, carry disproportionate, class-dependent predictive weight, we demonstrate that discriminative information resides where volume conduction is least homogenized. Furthermore, temporal dynamics contribute uniquely to separability. Waveform complexity (line length) consistently distinguished CFN from TN responses, with lower complexity strongly associated with the CFN and higher complexity driving TN classification. Together, these findings suggest that CDPs encode afferent identity in a tightly coupled spatiotemporal manifold, and that compressing this data into a lower-dimensional space inherently discards functionally critical structure.

The extensive anatomical overlap of lower-limb afferent projections within the lumbosacral enlargement produces complex, converging boundaries in feature space^41–43^. While establishing a linear baseline (LDA) yielded a robust median classification accuracy of 80.2% ± 0.3%, it failed to fully resolve these overlapping representations. The cubic kernel SVM captured this underlying nonlinear structure by projecting inputs into a higher-dimensional space, successfully mapping the entangled manifolds and significantly improving median classification accuracy to 90.9% ± 0.3%. While such nonlinear models maximize predictive performance, their mathematical complexity traditionally obscures physiological insight.

Therefore, integrating SHAP provided a critical feature-level attribution framework, linking model decisions directly to specific spatial and temporal components of the CDP and enabling the mechanistic interpretation of decoding in physiologically meaningful terms^44–46^.

When the Supra-MT-trained model was evaluated across lower stimulation intensities, it exhibited a graded reduction in overall accuracy (from a median of 90.9% ± 0.3% at Supra-MT, down to 65.0% at MT and 46.0% at Sub-MT), tracking with the progressive attenuation of evoked signal amplitude. Interestingly, when evaluating the cross-condition performance degradation, the signal attenuation was functionally asymmetric; CFN classification was highly sensitive to reduced intensity (falling to a 13.9% true positive rate for left CFN and 29.3% for right CFN at Sub-MT), whereas TN representations remained more robust (retaining 59.6% and 76.8% true positive rates for left and right TN, respectively). This divergence suggests a fundamental difference in the spatial and temporal encoding robustness of these specific afferent pools under conditions of reduced peripheral recruitment.

Crucially, however, when independent models were trained exclusively on these lower-intensity datasets, median classification accuracy recovered significantly to 81.6% ± 0.4% at MT and 64.5% ± 0.6% at Sub-MT. Notably, this recovered performance was symmetric across all four afferent classes, indicating that CFN information was not inherently lost. This supports the hypothesis that afferent-specific informational structures are preserved within low-amplitude, sub-perceptual CDPs; rather than degrading completely, the neural representations undergo nonlinear, intensity-dependent shifts in state space. Because the underlying afferent signature appears to be preserved and decodable, this suggests a viable pathway for continuous, background sensory state estimation in closed-loop systems without requiring stimulation above motor threshold.

Current clinically available closed-loop spinal cord stimulation systems primarily rely on evoked compound action potential amplitude as a feedback signal for stimulation control^35,47–49^. While effective for amplitude stabilization, this approach does not provide information about afferent signals, only the neural activation from the stimulus^50^. The present findings demonstrate that epidural recordings contain sufficient structured information to resolve afferent source identity even under reduced signal conditions. Combined with interpretable nonlinear decoding, this supports a transition from amplitude-based control to source-aware neural decoding. This framework establishes a basis for bidirectional spinal interfaces in which stimulation and afferent decoding are integrated within a single clinically viable epidural platform.

Determining if distinct afferent representations can be resolved at the dural surface is a prerequisite for epidural-based closed-loop systems that rely on real-time sensory state estimation to optimize therapeutic stimulation parameters. A critical advantage of this framework is that it operates entirely within the footprint of clinically available hardware. Demonstrating that standard 32-contact epidural paddle arrays can resolve fine-scale, source-specific spatiotemporal gradients, provides a practical substrate for future closed-loop systems without altering the established surgical workflow.

Furthermore, the ability to extract class-specific information from low-amplitude, threshold-dependent responses suggests that meaningful sensory decoding does not require massive, artificially synchronized volleys. This structural persistence implies that native proprioceptive or cutaneous traffic could eventually be decoded from the epidural space to drive adaptive, simultaneous stimulation and decoding.

While these findings establish a robust framework for epidural decoding, future clinical integration must address specific physiological and hardware variables. First, this study was conducted in a small cohort (N=3) of individuals with chronic, motor- and sensory-complete SCI. While this population provides essential experimental stability, chronic injury structurally alters local spinal excitability and conduction properties^51,52^. Future work should evaluate how this decoding framework generalizes across larger, heterogeneous cohorts, including incomplete injuries and neurologically intact individuals.

Second, epidural recordings reflect volume-conducted activity and inherently lack single-fiber resolution. Spatial localization is therefore constrained by cerebrospinal fluid filtering, electrode geometry, and our reliance on two-dimensional fluoroscopic reconstruction rather than direct anatomical imaging (e.g., post-operative CT-MRI fusion)^53–55^. While this imaging resolution may partially explain the model’s minimal reliance on the geometric features, the robust performance of the uncompressed feature space demonstrates that sufficient afferent specificity is preserved in the local voltage gradients despite these macroscopic blurring effects.

Finally, while this study establishes a vital computational baseline using controlled peripheral nerve stimulation in the supine resting condition, clinical neuromodulation typically occurs in dynamic behaviorally relevant settings. Translating this pipeline into real-time, closed-loop systems will require evaluating decoding stability during active motor tasks, where epidural signals are subjected to shifting cerebrospinal fluid dynamics and physiological artifacts. This translation will ultimately rely on integration with advanced implantable hardware to systematically assess computational latency, power constraints, and ambulatory robustness.

## Conclusion

Addressing a critical requirement for bidirectional neuroprostheses, this study provides the first-in-human demonstration that high-density epidural paddle arrays, combined with nonlinear and interpretable machine learning, can decode highly convergent lower-limb CDPs with a median cross-validated accuracy of 90.9% ± 0.3%. SHAP-based feature attribution and paired permutation testing reveal that this decoding is driven by fine-scale contact-level voltage patterns and temporal waveform complexity rather than derived geometric features, demonstrating that recorded epidural fields preserve distinct, source-specific afferent signatures. Furthermore, we show that these afferent-specific signatures persist even at sub-MT stimulation intensities, undergoing nonlinear, intensity-dependent shifts in state space rather than an inherent loss of information. Together, these findings establish a critical framework for moving beyond amplitude-based spinal cord stimulation toward source-aware, interpretable closed-loop neuromodulation using clinically deployed epidural interfaces.

## Methods

To map and classify the spatiotemporal structure of lower-limb afferent CDPs, we developed a three-stage neurophysiological and computational pipeline: (1) high-density epidural recordings during controlled peripheral nerve stimulation, (2) anatomically grounded spatial normalization of epidural paddle geometry using fluoroscopic reconstruction, and (3) multiscale feature extraction followed by interpretable linear (LDA) and nonlinear classification using a SVM with SHAP.

### Signal processing

Continuous recordings were processed in MATLAB (R2024b, MathWorks, MA). Signals were high-pass filtered (1 Hz, zero-phase, 2nd order Butterworth), notch filtered (60 Hz; zero-phase, 2nd order Butterworth) and segmented into stimulus-locked epochs using hardware triggers.

Next, a 15–750 Hz bandpass filter was applied prior to analysis. To reduce global contamination while preserving spatial structure, a participant-specific local reference was defined as the channel exhibiting the lowest mean root mean square (RMS) amplitude across all trials and stimulation conditions

(2) $$c_{\text{ref}}=\text{arg}\;{\text{min}}_{c}\frac{1}{K}\sum_{k=1}^{K}RMS_{c,k}$$

where the RMS at contact *c* is averaged over all *k* trials and *c*_*ref*_ is the contact that produced the lowest response across conditions for that participant. Finally, a strict artifact rejection procedure excluded trials in which ≥50% of channels exceeded 1.5×IQR of RMS energy (10–40ms window). To verify that this exclusion process did not introduce class imbalance, a Chi-square goodness-of-fit test was conducted on the retained trials to confirm a uniform distribution across the four afferent targets prior to model training.

### Machine learning and classification

An SVM was trained to classify four afferent targets (left/right CFN and TN)^56^. To ensure scale invariance while strictly preventing data leakage, feature standardization (z-scoring) was applied dynamically within each cross-validation fold. Specifically, standardization parameters were calculated exclusively from the pooled training partition (combining trials across participants for a tested intensity) and subsequently applied to the isolated testing partition. To capture the complex, overlapping spatial representations of these afferent targets, a cubic kernel SVM was used

(3) $$K(x_{i},\;x_{j})={\left(x_{i}x_{j}+1\right)}^{3}$$

where model regularization was set to *C = 1*, with automatic kernel scaling (heuristic optimization)^57^. Classification performance was evaluated using pooled, stratified 10-fold cross-validation (CV), and metrics included overall accuracy, true positive rate (TPR), false negative rate (FNR), positive predictive value (PPV), false discovery rate (FDR), and area under the receiver operating characteristic curve (ROC-AUC).

To evaluate the necessity of these nonlinear decision boundaries, we implemented a linear classification baseline model. We utilized a Linear Discriminant Analysis (LDA) model, integrated within the identical Error-Correcting Output Codes multi-class framework used for the primary SVM evaluation. Because our 42-dimensional feature space incorporates voltage patterns from high-density epidural contacts, the inherent multicollinearity of the data can render standard covariance matrix inversion mathematically unstable. For this reason, the LDA was configured to utilize a Moore-Penrose pseudo-inverse calculation to ensure the generation of a robust, strictly linear decision boundary.

To account for partition variance and ensure the robustness of the models, a repeated cross-validation approach (100 iterations of 10-fold CV) was employed to establish the median overall accuracy and standard deviation. Furthermore, to evaluate the performance degradation between the full spatiotemporal feature space and the restricted geometric feature model, a paired prediction permutation test was conducted. Out-of-fold trial predictions from a locked 10-fold partition were randomly swapped across 10,000 iterations to construct a null distribution, yielding the non-parametric *p*-value for classifier comparison.

### Model interpretability

To quantify feature-level contributions to classification performance within the non-linear SVM framework, SHAP was applied, enabling the explicit identification of spatial and temporal CDP features most strongly associated with the afferent source identity^27,28^.

## Supplementary Material

1

## Data Availability

Source data are provided with this paper.

## Code availability

All software and code used to generate the figures in this manuscript are available upon reasonable request to the corresponding author.
