# Supplementary material for: Decoding Overlapping Lower-Limb Afferent Pathways from Human Epidural Spinal Recordings
Source: Res Sq. 2026 Jul 22:rs.3.rs-10348238. Preprint. [Version 1] doi: 10.21203/rs.3.rs-10348238/v1 (PMC13419598; doi:10.21203/rs.3.rs-10348238/v1)
Supplement: 1 [file NIHPPRS10348238V1-supplement-1.pdf]

Supplementary Materials

Supplementary Table 1. - Descriptive spatiotemporal features of epidurally recorded cord dorsum potentials at supra-motor threshold intensities. *Data are presented as the median ± standard deviation for latency (ms), maximum peak-to-peak amplitude (Max Amp; µV), and temporal waveform complexity (Line Length, µV) across all three participants (P1-P3). Feature metrics are segmented by the four peripheral afferent stimulation targets: left and right common fibular nerves (LCFN, RCFN) and left and right tibial nerves (LTN, RTN). All metrics were derived from the epidural contact eliciting the maximal evoked response within the 10–40ms post-stimulus window during supra-motor threshold (Supra-MT) stimulation.*

	**P1**	**P2**	**P3**
		**LCFN**	
Latency (ms)	19.99 ± 8.79	14.53 ± 2.56	18.86 ± 1.87
Max Amp (µV)	16.24 ± 11.41	23.39 ± 2.36	28.59 ± 2.93
Line Length (µV)	166.58 ± 23.20	99.04 ± 8.07	107.95 ± 7.72
		**RCFN**	
Latency (ms)	15.63 ± 4.23	14.42 ± 4.26	21.22 ± 3.01
Max Amp (µV)	16.24 ± 11.41	19.79 ± 3.15	23.57 ± 3.06
Line Length (µV)	130.33 ± 16.04	93.83 ± 8.37	121.11 ± 9.82
		**LTN**	
Latency (ms)	24.75 ± 7.31	20.14 ± 0.36	24.34 ± 8.90
Max Amp (µV)	14.33 ± 2.84	24.02 ± 3.23	26.77 ± 9.38
Line Length (µV)	156.19 ± 25.56	103.06 ± 9.56	152.59 ± 31.19
		**RTN**	
Latency (ms)	23.80 ± 8.08	20.64 ± 2.69	24.62 ± 8.99
Max Amp (µV)	14.68 ± 4.30	17.50 ± 2.88	35.76 ± 8.69
Line Length (µV)	156.70 ± 28.19	95.14 ± 10.75	225.88 ± 37.26

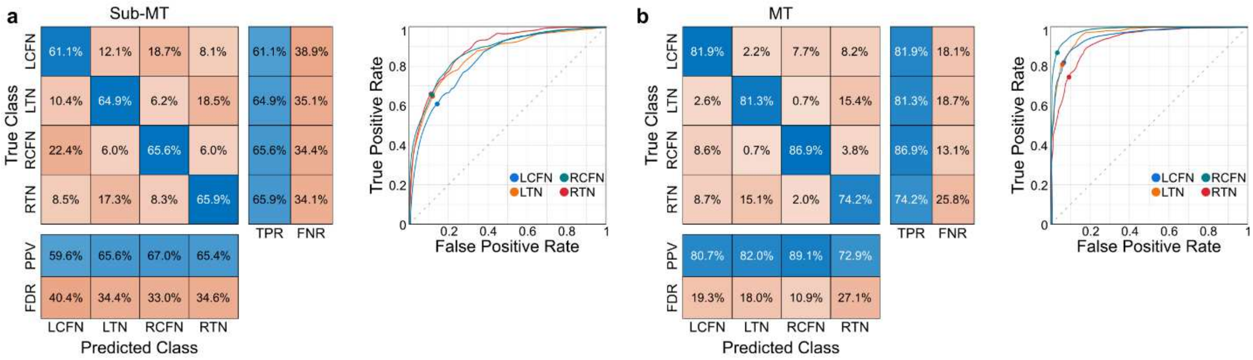

Supplementary Figure 1 - Nonlinear decoding recovery at lower stimulation intensities. *Independent cubic kernel SVM models were trained and evaluated exclusively on datasets from lower stimulation intensities to assess the preservation of afferent-specific structure.* ***a.*** *Cross-validated confusion matrix and corresponding receiver operating characteristic (ROC) curves for a representative sub-motor threshold (Sub-MT) model. The model recovered an overall classification accuracy of 64.4% and maintained class separability (AUC: 0.836–0.888).* ***b.*** *Cross-validated confusion matrix and ROC curves for a representative motor threshold (MT) condition, achieving an overall accuracy of 81.4% (AUC: 0.925–0.983). Unlike the fixed-boundary Supra-MT model applied to the Sub-MT and MT data in the main text, these independently trained models confirm that afferent spatial topographies undergo nonlinear, intensity-dependent shifts while remaining fundamentally decodable.*

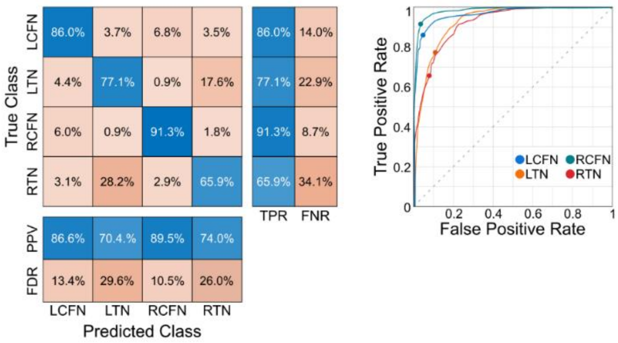

Supplementary Figure 2 - Linear decoding baseline of overlapping afferent sources. *A Linear Discriminant Analysis (LDA) model was trained on the 42-dimensional spatiotemporal feature space derived from Supra-MT stimulation to establish a baseline for spatial separability. The cross-validated confusion matrix (left) for a representative model demonstrates an overall classification accuracy of 82.0%. While the ROC curves (right) indicate strong separability across classes (AUC: 0.920–0.982), the linear model failed to fully resolve the overlapping afferent representations compared to the cubic kernel SVM (92.0% representative; 90.9% median), highlighting the necessity of nonlinear decision boundaries to map the complex, volume-conducted epidural fields.*
